# Pathogen identification and biological fungicides screening for *Plumbago auriculata* blight in China

**DOI:** 10.3389/fmicb.2025.1609944

**Published:** 2025-07-04

**Authors:** Ming Liu, Tiantian Guo, Hao Yan, Yue Yuan, Zhien Xiao, Yuxin Liu, Shaotian Zhang, Fengqing Lyu, Shan Jing, Fuqiang Yin

**Affiliations:** ^1^College of Biological and Food Engineering, Chongqing Three Gorges University, Chongqing, China; ^2^The Chongqing Engineering Laboratory for Green Cultivation and Deep Processing of the Three Gorges Reservoir Area’s Medicinal Herbs, Chongqing, China; ^3^Chongqing Wanzhou Productivity Promotion Center, Chongqing, China

**Keywords:** *Plumbago auriculata*, *Fusarium ipomoeae*, blight, pathogen, multigene phylogeny, biological fungicides screening, control efficacy

## Abstract

*Plumbago auriculata* is an important ornamental horticultural plant with high ornamental value. *Plumbago auriculata* blight was first detected in 2023 in Wanzhou District, Chongqing City, China. This disease seriously reduces the ornamental value of *P. auriculata*. The disease was characterized by the yellowing and drying up of the apex in the early stage and the drying up and death of the entire aboveground part in the later stage. To identify the pathogenic fungus of *P. auriculata* blight in Wanzhou district of Chongqing and to screen effective biological pesticides for controlling the disease, the pathogen was isolated and cultured using the tissue separation method. The pathogens were identified by morphology combined with multigene analysis. Cross-pathogenicity experiments were conducted on two other horticultural plants using the pathogen. Biological fungicides were screened by an indoor toxicity test. Combined with the potted plant prevention effect experiment, the control efficacy of the biological fungicide was evaluated. The results showed that isolates L9 and L11 colonies have white cotton flocculent aerial mycelium. The macroconidia are falcate, prominently cell papillate, and hooked. Numerous chlamydia spores were observed through PDA. L9 and L11 were identified by phylogenetic analysis (internal transcribed spacers, RNA polymerase II second largest subunit, translation elongation factor 1 alpha, and calmodulin) and clustered together with *Fusarium ipomoeae* in the same single clade. This is the first report that *F. ipomoeae* causes blight on *P. auriculata* in China. *Fusarium ipomoeae* was pathogenic to *Prunus serrulata* and *Heptapleurum arboricola*. The results of the indoor toxicity test showed that the inhibitory effect of 0.4% osthole SL on *F. ipomoeae* was significant, with an EC_50_ value of 1.089 μg/mL. 0.4% osthole SL has a good prevention and control effect on *P. auriculata* blight, with a control efficacy of 88%. Osthole can be used for the prevention and control of *P. auriculata* blight. The results provided the foundation for the recognition and green control of *P. auriculata* blight caused by *F. ipomoeae*.

## 1 Introduction

*Plumbago auriculata* (*P. auriculata*) is an erect perennial herb in the family Plumbaginaceae and the genus Plumbago. *Plumbago auriculata* is native to South Africa and has been widely introduced as an ornamental plant in various countries (Shen et al., 2021). It is a commonly used plant in urban landscaping (Vyapari et al., 2007; Ning et al., 2011). With the increase of planting area, the occurrence of *P. auriculata* diseases has become common. However, there are few reports on *P. auriculata* disease. Only leaf yellowing in *P. auriculata* caused by *Candidatus Phytoplasma asteris* has been reported (Panda et al., 2019).

Blight is a devastating disease of plants. *Fusarium* spp. (Pandey et al., 2024; Mohamed et al., 2021)*, Verticillium dahlia* (Hassan et al., 2023)*, Paramyrothecium* spp. (Kumar et al., 2025)*, Plectosphaerella* spp. (Yang et al., 2023)*, Lasiodiplodia* spp. (Kwon et al., 2017), *Botryosphaeria* spp. (Rui et al., 2025) can all cause blight. In 2023, a large area of *P. auriculata* was found in Wanzhou District, Chongqing, China, with chlorosis of leaf stem and browning of branch vascular bundle, which was similar to the typical symptoms of blight. However, the pathogen that causes the blight of *P. auriculata* is unclear.

*Fusarium* spp. is a prevalent pathogenic fungus that infects many hosts and is the primary causal agent of wilt, root rot, and leaf spot. Examples include acacia seedling wilt disease (Soleha et al., 2022), foliar blight on *Begonia semperflorens* (Lin et al., 2023), chrysanthemum wilt (Balamurugan et al., 2024), root rot in *Fatsia japonica* (Xu et al., 2024), root rot of Hydrangea (Neupane et al., 2023), leaf spot of *Hosta ventricosa* (Wang et al., 2021). Often called “plant cancer,” *Fusarium* wilt disrupts the vascular system (Yao et al., 2025). There have been no reports of *Fusarium* spp. causing disease on *P. auriculata*.

In this study, the pathogenic fungi of *P. auriculata* blight found in the Wanzhou district of Chongqing were isolated and purified. A multi-gene combined phylogenetic tree was constructed using internal transcribed spacers (ITS), RNA polymerase II second largest subunit (*RPB2*), translation elongation factor 1 alpha (*EF–1α*), and calmodulin (*CAMD*), and the pathogenic fungi were identified by combining morphology. Cross-pathogenicity experiments were conducted on two other horticultural plants using the pathogen. Through indoor toxicity tests, suitable biopesticides were selected. Combined with the potted plant prevention effect experiment, the control efficacy of the biological fungicide was evaluated. This study aimed to identify the pathogenic fungus causing *P. auriculata* blight in Wanzhou district of Chongqing, select effective biological fungicides for better prevention and control effects, and provide a reference for the identification and targeted control of *P. auriculata* blight.

## 2 Materials and methods

### 2.1 Sample collection and fungal isolation

In 2023, a total of 20 leaves and stems of blight on *P. auriculata* with typical symptoms were collected in Wanzhou District (108°26.4′N,30°45′E), Chongqing City, China. Wash with running water, dry naturally. The disease samples of 5 mm × 5 mm at the junction of disease and healthy were cut with a sterile scalpel, surface sterilized in 75% ethanol for 1 min and then in 3% NaClO for 4 min, rinsed three times in sterilized distilled water. Air-dried on sterilized filter paper, the samples were then placed on antibiotic (streptomycin sulphate, 50 μg/mL) amended potato dextrose agar (PDA) plates and incubated for 5 days at 25°C (Lyu et al., 2024). According to the characteristics of the colony and morphological characteristics, several representative strains were purified and cultured at 25°C on PDA. Inoculate the isolates onto a PDA tube ramp and store at 4°C (Wang et al., 2022).

### 2.2 Pathogenicity tests

*In vitro* inoculation was carried out using the mycelium plugs inoculation method and the conidia suspension inoculation method to inoculate detached leaves and stems, respectively (Tang et al., 2022; Ao et al., 2024). *In vivo* inoculation was carried out using the conidia suspension inoculation method. Inoculate healthy *P. auriculata* potted plants (Tang et al., 2022).

Prior to *in vitro* inoculation, rinse the healthy leaves and stems with 75% alcohol for 1 min, wash them with 0.4% sodium hypochlorite solution for 30 s, and then rinse them three times with sterile water (each time for 1 min). Dry naturally. On a healthy leaf, prick the leaf symmetrically with a sterilized needle (the main leaf vein is symmetrical on both sides). The mycelium plugs were dispensed from the representative strain L9 with a sterile punch (5 mm in diameter) and placed in *P. auriculata* healthy leaves flanking the main veins. Another healthy leaf was taken and inoculated with sterile potato dextrose agar medium as a control. After culturing the representative strain L9 on PDA for 7 days, a 1 × 10^6^ mL conidia suspension was prepared. Spray conidia suspension (1 × 10^6^ conidia/mL) onto *P. auriculata* healthy stems while using the same procedure with sterile distilled water as a control. All inoculated leaves and stems were placed in 25°C Petri dishes covered with moist sterile filter paper (80% relative humidity).

The potted plants used for *in vivo* inoculation were healthy *P. auriculata* that were 3 months old. Before *in vivo* inoculation, disinfect the surface of the *P. auriculata* plants with 75% alcohol, rinse them with sterile water, and allow them to dry naturally. Inoculate *P. auriculata* plants with a conidia suspension by applying an average of 5 mL of conidia suspension (1 × 10^6^ conidia/mL) to each plant until the plant surfaces are completely moist. Healthy plants sprayed with sterile distilled water served as negative controls.

The inoculated materials were placed in a temperature incubator and cultivated at 25°C, with a photoperiod of 12 h and a relative humidity (RH) of 80%. Both the *in vitro* inoculation experiment and the *in vivo* inoculation experiment comprised five repetitions. To observe whether the disease symptoms of plants in the temperature incubator were consistent with the field symptoms regularly. After typical symptoms appeared in stems and leaves, the pathogenic fungus was reisolated from the infected leaves and stems. If the isolated strain was consistent with the inoculated strain, it was identified as the pathogen.

### 2.3 Morphological characterization

Two representative isolates (L9 and L11) obtained were cultured on potato dextrose agar (PDA) at 25°C in the dark for 5 days to observe the morphological characteristics of colonies by optical microscope. Preliminary identification of species was based on morphological characteristics of the pathogenic strains on PDA, including colony color, texture, pigment production, and the morphology of conidiophores and chlamydospores. For each representative fungal isolate, 50 conidia and chlamydospores were randomly selected for measurement.

### 2.4 Molecular identification and phylogenetic analysis

Representative fungal strains were cultured, and mycelium was collected. Plant genomic DNA extraction kit (Cwbio, Jiangsu, China) was used to extract DNA representing strains L9 and L11. Primers ITS1/ITS4 (White et al., 1990), *5F2/7CR* (Reeb et al., 2004), *EF1/EF2* (O'Donnell et al., 1998), *CL1/CL2A* (O’Donnell et al., 2000) were used to amplify four loci of L9 and L11, including the internal transcribed spacers (ITS), RNA polymerase II second largest subunit (*RPB2*), translation elongation factor 1 alpha (*EF–1α*), and calmodulin (*CAMD*).

The PCR amplification reaction system was 20 μL, including Taq Master Mix (K1071, Sangon Biotech (Shanghai) Co. Ltd) 10 μL, 10 μmolL^−1^ upstream and downstream primers 0.3 μL each, DNA template 2 μL, and ddH_2_O added to 20 μL. The amplification conditions used for the ITS1/ITS4, *5F2/7CR, EF1/EF2*, *CL1/CL2A*, and primer pairs are illustrated in Table 1. After amplification, part of the reactants was detected by 1% agarose gel electrophoresis. The remaining reactants were sent to Sangon Biotech (Shanghai) Co., Ltd. (Chengdu) for sequencing. The obtained sequences were compared and analyzed using GenBank^1^ to download high-consistency sequences (Table 2). These sequences were combined in PhyloSuite to form the ITS-*RPB2*-*EF–1α*-*CAMD* sequence, and a phylogenetic tree was constructed using the Maximum Likelihood method in MEGA11.0 software, with the repetition value set to 1,000 times. The obtained DNA sequences were uploaded to the National Center for Biotechnology Information to acquire accession numbers.

**Table 1:** Primer pairs, PCR amplification, and procedures used in this study.

Gene	Primer		PCR amplification procedures
	Primer code	Sequence (5′-3′)	
ITS	ITS1	TCCGTAGGTGAACCTGCGG	94°C 5 min; 35 cycles of 94°C 45 s, 55°C 45 s, 72°C 1 min; 72°C10 min
	ITS4	TCCTCCGCTTATTGATATGC	
*RPB2*	*5F2*	GGGGWGAYCAGAAGAAGGC	95°C 5 min; 35 cycles of 95°C 30s, 56°C 30 s, 72°C 1 min; 72°C 10 min
	*7CR*	CCCATRGCTTGYTTRCCCAT	
*EF–1α*	*EF1*	ATGGGTAAGGARGACAAGAC	94°C 5 min; 35 cycles of 94°C 45 s, 56°C 45 s, 72°C 2 min; 72°C 10 min
	*EF2*	GGARGTACCAGTSATCATG	
*CAMD*	*CL1*	GARTWCAAGGAGGCCTTCTC	94°C 90 s; 36 cycles of 94°C 45 s, 55°C 45 s, 72°C 1 min; 72°C 10 min
	*CL2A*	TTTTTGCATCATGAGTTGGAC	

**Table 2:** GenBank accession number used in the phylogenetic tree.

Species	Strain number	Host	Location	ITS	*RPB2*	*EF*	*CAMD*
***F. ipomoeae***	**L9**	***Plumbago auriculata***	**China**	**PV469435**	**PV273242**	**PP971762**	**PP971764**
***F. ipomoeae***	**L11**	***Plumbago auriculata***	**China**	**PV470910**	**PV053561**	**PP957444**	**PP971763**
*F. acuminatum*	NJC24	*Allium sativum*	China	OL655401	OL741723	OL741713	–
*F. acuminatum*	NJC23	*Allium sativum*	China	OL655400	OL741720	OL741722	–
*F. ananatum*	CBS 118517	*Ananas comosus*	South Africa	–	MN534229	MN533988	MN534157
*F. ananatum*	CBS 118518	*Ananas comosus*	South Africa	–	MW402730	MW401979	MW402377
*F. arcuatisporum*	LC12147	*Brassica campestris*	China	MK280802	MK289739	MK289584	MK289697
*F. arcuatisporum*	LC6026	*Nelumbo nucifera*	China	MK280792	MK289770	MK289585	MK289667
*F. commune*	FBG2020_198	*Zinnia elegans*	American	MT973967	–	MW020579	–
*F. commune*	FBG2020_199	*Zinnia elegans*	American	MW018368	–	MW020577	–
*F. guilinense*	NRRL 13335	*Medicago sativa*	Australia	GQ505679	GQ505768	GQ505590	GQ505502
*F. guilinense*	LC12160	*Musa nana*	China	MK280837	MK289747	MK289594	MK289652
*F. ipomoeae*	GZAX 307	*Nicotiana tabacum*	China	ON961779	ON982725	ON982723	ON982721
*F. languescens*	CBS 645.78	*Solanum lycopersicum*	Morocco	–	MH484880	MH484971	MH484698
*F. languescens*	CBS 413.90	*Solanum lycopersicum*	Israel	–	MH484890	MH484981	MH484708
*F. mangiferae*	Iso5	*Mangifera indica*	Pakistan	OQ179789	–	OQ184927	–
*F. mangiferae*	Iso4	*Mangifera indica*	Pakistan	OQ179788	–	OQ184926	–
*F. nanum*	LC1385	*Solanum lycopersicum*	Saudi Arabia	MK280781	MK289765	MK289612	MK289662
*F. nanum*	LC1516	*Solanum lycopersicum*	Saudi Arabia	MK280782	MK289766	MK289613	MK289663
*F. nirenbergiae*	Di3A-Pef 5	*Passiflora edulis*	Italy	MZ398145	MZ408113	MZ408118	–
*F. nirenbergiae*	Di3A-Pef 4	*Passiflora edulis*	Italy	MZ398144	MZ408112	MZ408117	–
*F. ophioides*	CBS 118512	*Panicum maximum*	South Africa	–	MN534303	MN534022	MN534209
*F. ophioides*	CBS 118513	*Panicum maximum*	South Africa	–	MN534300	MN534023	MN534202
*Alternaria alternata*	REIS 68	*Ceratostigma willmottianum*	Italy	MN565914	MN566300	MN627329	MK558222

The bold values indicate the test strains used in this study.

### 2.5 Cross-pathogenicity test

Healthy detached leaves were inoculated by mycelium plug inoculation to determine whether the representative strain L9 was also pathogenic to other horticultural plants (Ao et al., 2024). The selected two horticultural plants are *Prunus serrulata* Lindl. (which belongs to the family Rosaceae and the genus Prunus L.) and *Heptapleurum arboricola* Hayata (which belongs to the family Araliaceae and the genus *Schefflera*).

The mycelium plugs inoculation method was carried out as described in section 2.2. The experiment comprised five repetitions. The inoculated leaves were placed in a temperature-controlled incubator and cultivated at 25°C, with a 12-h photoperiod and 80% relative humidity (RH). When re-isolating the pathogen, it matched the pathogen from leaves with lesion symptoms. If the re-isolate was consistent with isolate L9, it confirmed that L9 was pathogenic to *P. serrulata* and *H. arboricola*.

### 2.6 Fungicide assays

The inhibitory effect of seven types of biological pesticides on pathogenic fungi was measured using the mycelium growth rate method. Each biological pesticide was made into pharmaceutical solutions with different concentration gradients using sterile water (Table 3). The different pharmaceutical solutions concentrations were added to the sterilized PDA medium according to a certain volume ratio. The mixture was thoroughly mixed, and medicine plates were prepared with different test concentrations. Each treatment was repeated five times for each concentration. A sterile punch (5 mm in diameter) was used to extract the mycelium plugs of a representative strain L9 at the edge of the colony. The mycelium plugs were inoculated in the center of the medicine-prepared plates. PDA medium with an equal amount of sterile water served as a control. The plates were incubated at 25°C in darkness within a constant temperature incubator for 5 days. The colony diameter was measured using the cross-shaped intersecting method.

**Table 3:** Pharmaceutical name and test concentrations.

Pharmaceutical name	Test concentrations (μg/mL)
0.4% Osthole SL	20.000, 5.000, 2.500, 1.250, 0.625, 0.313
1% Phenazino-1-carboxylic acid SC	50.000, 25.000, 12.500, 6.250, 3.125, 1.563
80% Ethylicin EC	120.000, 60.000, 30.000, 15.000, 7.500, 3.750
5% Avermectin EC	160.000, 80.000, 40.000, 20.000, 10.000, 5.000
3% Zhongshengmycin AS	500.000, 250.000, 125.000, 62.500, 31.250, 15.625
4% Berberine AS	500.000, 250.000, 125.000,6 2.500, 31.250, 15.625
8% Ningnanmycin AS	1000.000, 500.000, 250.000, 125.000, 62.500, 31.250

SL, soluble concentrate; SC, suspension concentrate; EC, emulsifiable concentrate; AS, aqueous solution.

The percent inhibition of mycelial growth (PIMG) was calculated using the following formula (where *F* is the diameter of the fungal plug, *C* is the radial growth diameter of the fungus in the control, and *T* is the radial growth diameter of the fungus in the treatment group) (Ao et al., 2024).


$$\text{PIMG}\;(\%)=\frac{C-T}{C-F}\times 100\%$$

Microsoft Excel 2019 was used to calculate a toxicity regression equation (where *x* is the logarithm of the fungicide concentration (μg/mL) and *y* is the inhibition rate) and correlation coefficient for each fungicide. SPSS 26.0 was used to calculate the half maximal effective concentration (EC_50_) value of each fungicide. We used ANOVA with post-hoc Duncan’s test in SPSS 26.0 to compare inhibition rates across treatments at different concentrations among the same fungicides. And mark the statistical differences with lowercase letters.

### 2.7 Potted plant prevention effect test

Based on the indoor toxicity test results, the fungicide 0.4% osthole SL, which showed an obvious antibacterial effect, was selected for the pot control effect test. The treatment was carried out with three concentration gradients of 0.4% osthole SL EC_50_, EC_70_, and EC_90_ (1.089 μg/mL, 2.071 μg/mL, and 5.244 μg/mL, respectively). The treatment without any pharmaceutical application, but inoculated with the isolate L9, served as the negative control. Each treatment was set up with five replicates.

After culturing the representative strain L9 on PDA for 7 days, 1 × 10^6^ mL of conidia suspension was prepared in advance. Sterilize the soil in the potted plants in advance. Select healthy *P. auriculata* plant seedlings of the same size and age of 3 months and transfer them into seedling pots. After they stand upright and grow normally, disinfect the surface of *P. auriculata* plants with 75% ethanol and rinse with sterile water. Inoculate an average of 5 mL of the conidia suspension per plant until the surface of the plants is completely moist. The potted plants were inoculated and sprayed with 0.4% osthole SL early in the disease for prevention and control. Fungicides were applied once every 7 days for a total of 4 times. Spray each plant with a micro sprayer to ensure even application of the medicine. The potted plants were placed in a greenhouse and cultivated at 25°C, with a photoperiod of 12 h and a relative humidity (RH) of 80%. The disease severity was investigated 1 day before the application of the pesticide and on the 7th day after the application ended, and the disease severity (DS) and control efficacy (CE) were calculated, respectively (Wang et al., 2024).

The DS was scored for each plant according to the percentage of leaves and stems with yellowing or necrosis where 0 = No evidence of lesions, 1 = 33% of leaf and stem area infected, 2 = 34–66% of leaf and stem area infected, 3 = 67–100% of leaf and stem area infected and 4 = dead plant (Díaz-Gutiérrez et al., 2021). The disease severity index (DSI) was calculated for each treatment using the formula of Promwee et al. (2017).


$$\mathrm{DSI}\;(\%)=(\frac{\left(\sum (\text{Scale}\times \text{Amount of plants}\right)}{\text{Maximum level}\times \text{Total plants}})\times 100$$

The control efficacy (CE) was calculated for each treatment using the formula of Wang et al. (2024) (where *D* is the disease severity index in the blank control area, and *d* is the disease severity index of the treatment area).


$$\mathrm{CE}=\frac{D-d}{D}\times 100\%$$

Data were collated using Microsoft Excel 2019 software, and the Duncan’s test in SPSS 26.0 statistical software was used for the significance analysis of differences.

## 3 Results

### 3.1 Symptom characteristics

**Figure 1:**
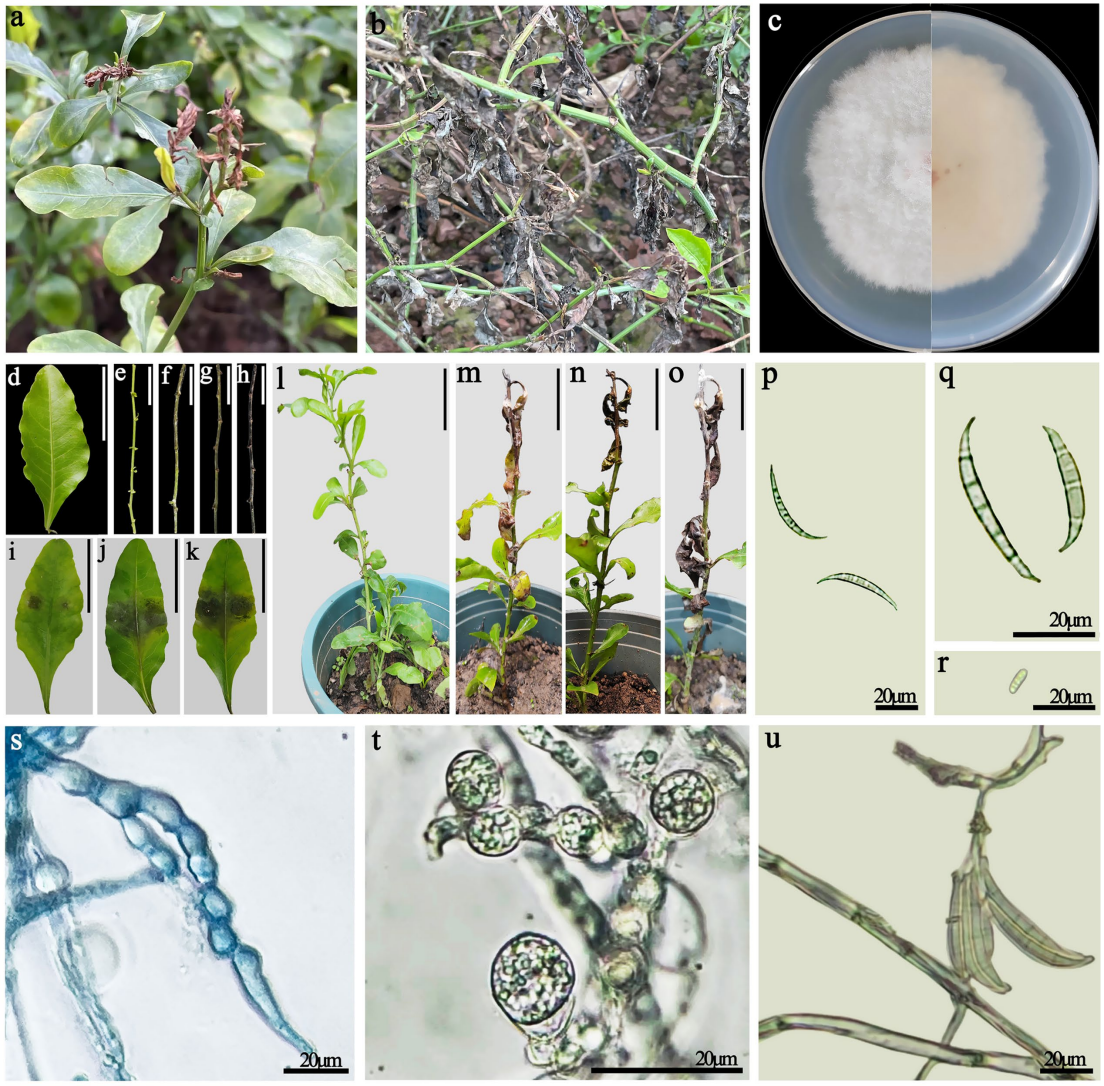
**(a,b)** Symptoms of disease in *Plumbago auriculata* in the field. **(a)** Early field diseases occur at the tip of the stem; **(b)** In the later stages of the disease, the aboveground parts died. **(c)** Colony morphology. **(d,e)** Negative controls, healthy detached leaves and stems. **(f–h)** Symptoms on healthy detached stems inoculated with *F. ipomoeae* (at 4, 8, and 12 days post-inoculation, respectively). **(i–k)** Symptoms on healthy detached leaves inoculated with *F. ipomoeae* (at 4, 8, and 12 days post-inoculation, respectively). **(l)** Negative controls, healthy potted plants of *P. auriculata*. **(m–o)** Symptoms on healthy potted plants inoculated with *F. ipomoeae* (at 5, 10, and 15 days post-inoculation, respectively). **(p,q)** Macroconidia. **(r)** Microconidia. **(s,t)** Chlamydospore. **(u)** Macroconidia form on the sporophore. **(d–o)** Scale bars = 50 mm.

According to the investigation, the incidence of wilt disease of *P. auriculata* was 50–60% in the field. The disease started in April; the worst onset was in September to October. The initial symptoms of the disease are the yellowing and wilting of the top stems and young leaves. The leaves lost water, curled, and the junction between disease and health appeared dark brown (Figure 1a). The symptoms gradually spread downwards. The leaves became dry, curly, drooping, and dying. The later symptoms were manifested as the withering and death of the entire plant (Figure 1b).

### 3.2 Morphological characteristics of the pathogen

Fungal colonies have white cotton flocculent aerial mycelium after 7 days of incubation under dark conditions on PDA. The back of the colony is pale pink. The edges of the colony are uneven (Figure 1c). The macroconidia are falcate, prominently cell papillate or hooked, and have 4–6 septa, measuring 40–68 × 4–7 μm (Figures 1p,q). Microconidia are oval and have either one or no septa. Microconidia 8–10 × 3–5 μm (Figure 1r). A lot of chlamydia spores are observed on PDA. Chlamydospores were produced in chains or pairs, globose, and thick-walled (Figures 1s,t). The conidiophores in the sporodochia vary in length and are branched verticillately. The hyphae are thin-walled and hyaline (Figure 1u). These characteristics suggest the pathogenic fungus was *Fusarium* spp.

### 3.3 Pathogenicity test

Four to five days after inoculation with the representative strain L9, the detached stems showed chlorosis and yellowing (Figure 1f). Brown spots were observed on the detached leaves (Figure 1i). Wilting and yellowing occurred in leaves and at the tips of the stems of *P. auriculata* potted plants (Figure 1m). After 8–10 days, the outer layer of the stems turned light brown (Figure 1g). The browning area of the detached leaves expanded, and some gradually died (Figure 1j). The leaves of potted plants have become dry and curly (Figure 1n). After 12–15 days, the outer layer of the detached stems turned dark brown (Figure 1h). The inoculated site of the detached leaves completely turned brown and withered (Figure 1k). The symptoms of withering in potted plants gradually spread downward, and the drooping leaves completely withered and died. White mycelial layers appeared on the surface of diseased plant tissues (Figure 1o). The boundary between diseased and healthy plant tissues appeared dark brown. The symptoms of the indoor pathogenicity test were consistent with those of the field diseases. In contrast, all the control groups remained healthy on the 15th day (Figures 1d,e,l).

The inoculated and diseased *P. auriculata* leaves and stems were subjected to pathogen re-isolation and identification. The re-isolated pathogens were consistent with the inoculated pathogens. Thus, we identified L9 as the pathogen causing blight in *P. auriculata*.

### 3.4 Molecular identification and phylogenetic analyses

**Figure 2:**
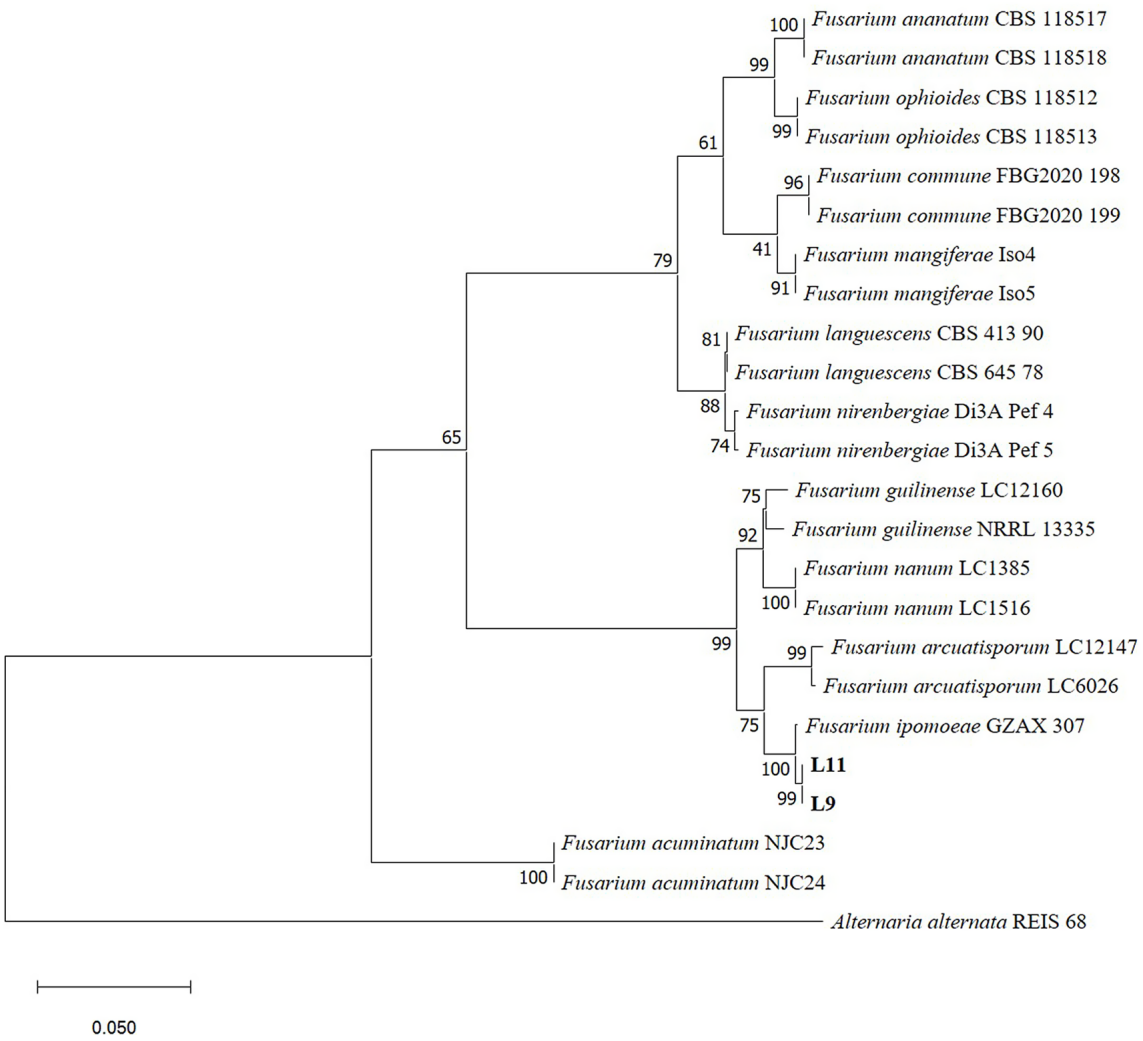
Phylogenetic tree calculated from the alignment of concatenated sequences of the internal transcribed spacer (ITS), RNA polymerase II second largest subunit (*RPB2*), translation elongation factor 1 alpha (*EF–1α*) and calmodulin (*CAMD*) genes using Maximum likelihood method. L9 and L11 are the isolates described in this study. Bootstrap values > 50% (1,000 replications) are given at the nodes. Bar = 0.05 substitution per nucleotide position. *Alternaria alternata* strain REIS 68 was used as an outgroup.

The gene regions of ITS, *RPB2*, *EF–1α* and *CAMD* were PCR amplified and sequenced. The obtained sequences of ITS, *RPB2*, *EF–1α* and *CAMD* were deposited in GenBank. The combined dataset of ITS, *RPB2*, *EF–1α* and *CAMD* genes was used for phylogenetic analysis by MEGA11.0. *Alternaria alternata* strain REIS 68 was used as an outgroup. The ML tree demonstrated that L9 and L11 isolates were placed in the *F. ipomoeae* (GZAX 307) group, supported by a 100% bootstrap value (Figure 2). Representative isolates L9 and L11 were confirmed as *F. ipomoeae* based on morphological characteristics and molecular identification.

### 3.5 Cross-pathogenicity test

**Figure 3:**
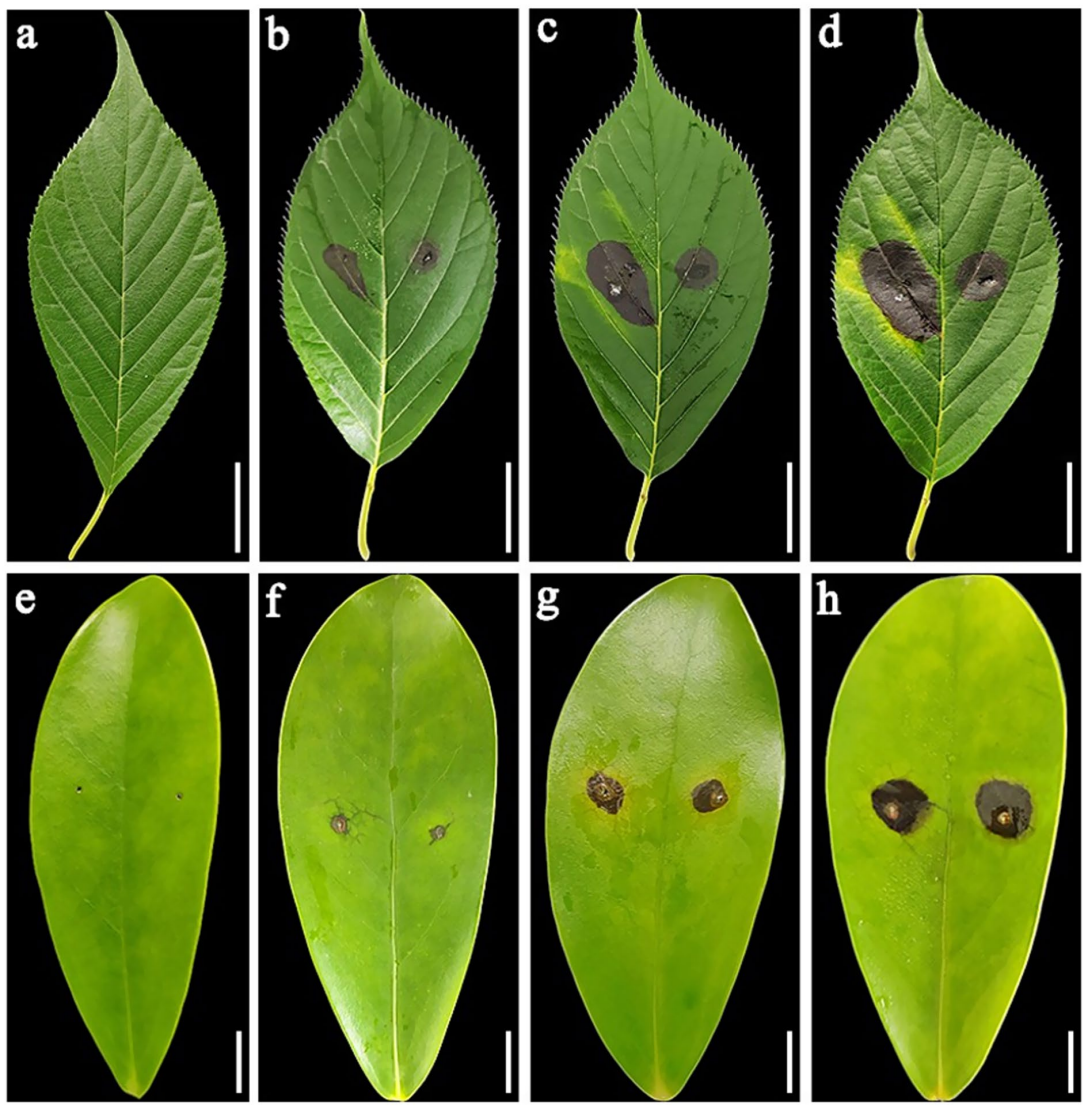
**(a)** Negative controls, healthy detached leaves of *Prunus serrulate*. **(b–d)** Symptoms on *Prunus serrulate* healthy detached leaves inoculated with *Fusarium ipomoeae* (at 4, 8, and 12 days post-inoculation, respectively). **(e)** Negative controls, healthy detached leaves of *Heptapleurum arboricola*. **(f–h)** Symptoms on *Heptapleurum arboricola* healthy detached leaves inoculated with *F. ipomoeae* (at 4, 8, and 12 days post-inoculation, respectively). **(a–d)** Scale bars = 20 mm. **(e–h)** Scale bars = 10 mm.

After *in vitro* inoculation of the isolated plant leaves of *P. serrulata* and *H. arboricola*, the inoculated parts of the plant leaves showed symptoms of disease to varying degrees. Strain L9 had a relatively strong pathogenicity to *P. serrulata*. Four days after inoculation, 3–4 mm brown small lesions appeared at the pinhole. After 12 days of inoculation, the lesion size reached 17–25 mm (Figures 3a–d). Conversely, the strain L9 had weaker pathogenicity against *H. arboricola*. Twelve days after inoculation, the size of the lesion was 9–11 mm (Figures 3e–h). Twelve days later, none of the control group showed any symptoms of the disease. Re-isolate the pathogen from the diseased leaves, and the resulting pathogen was consistent with that of L9. The isolate L9 was pathogenic to both *P. serrulata* and *H. arboricola*. *Fusarium ipomoeae* isolated from *P. auriculata* blight can infect other horticultural plants.

### 3.6 Fungicide assays

**Figure 4:**
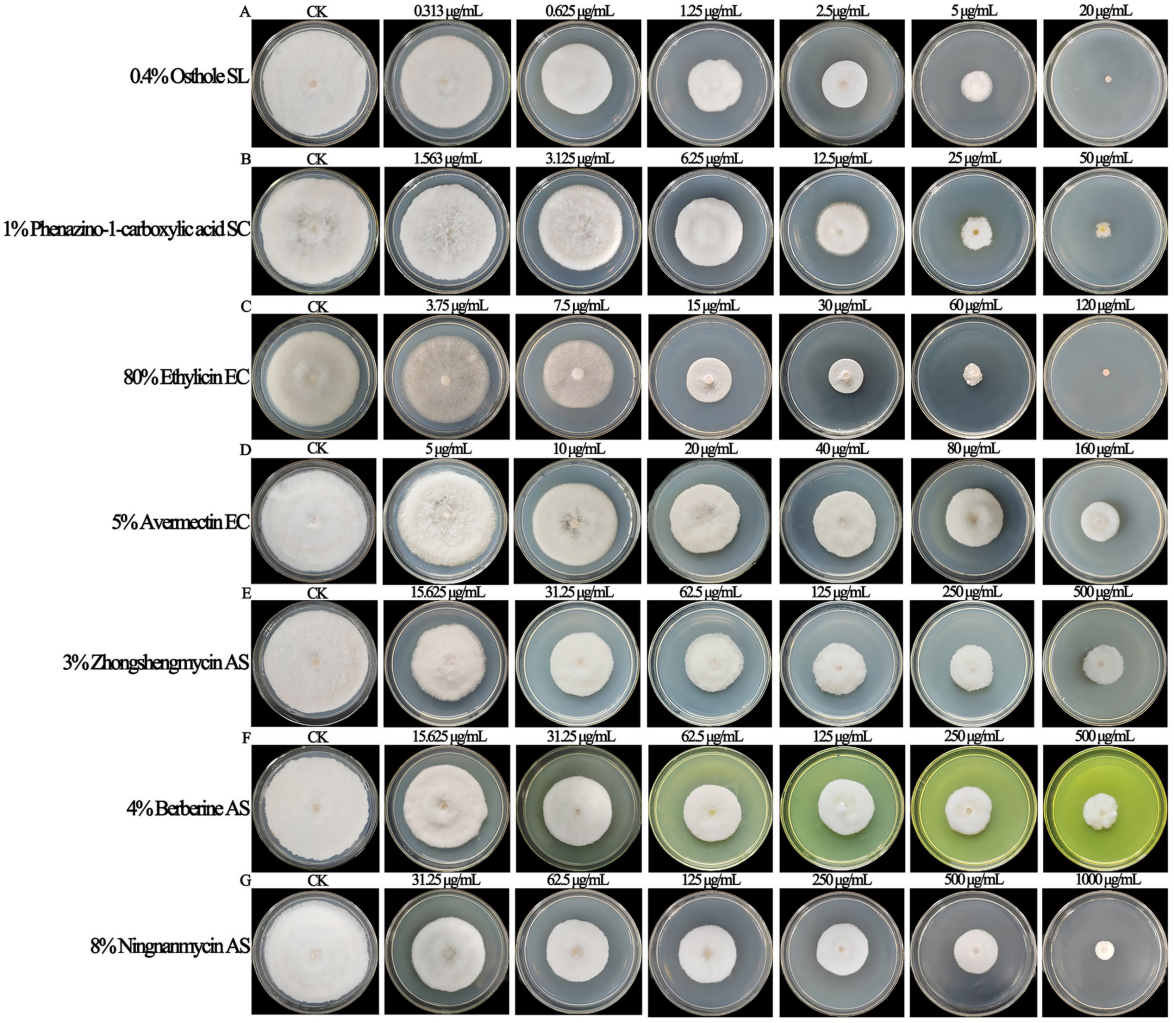
The biological pesticides treated with different fungicide types and concentrations could inhibit the mycelial growth of *Fusarium ipomoeae* to different degrees. **(A)** 0.4% Osthole SL; **(B)** 1% Phenazino-1-carboxylic acid SC; **(C)** 80% Ethylicin EC; **(D)** 5% Avermectin EC; **(E)** 3% Zhongshengmycin AS; **(F)** 4% Berberine AS; **(G)** 8% Ningnanmycin AS.

The biological pesticides treated with different fungicide types and concentrations could inhibit the mycelial growth of *F. ipomoeae* to different degrees (Figure 4). For each biopesticide used in the fungicide assays, the original fungicide concentration, formulation type, toxicity regression equation, correlation coefficient, EC_50_ value, and 95% confidence intervals are shown in Table 4.

**Table 4:** Sensitivity of 7 biological pesticides to pathogen *Fusarium ipomoeae.*

Pharmaceutical name	Test concentrations (μg/mL)	Bacteriostatic rate/%	Toxicity regression equation	Correlation coefficient (R^2^)	EC_50_/ (μg·mL^−1^)	Confidence interval of 95%
0.4% Osthole SL	20.000	99.69 ± 0.14^a^	y = 0.8869x + 4.9327	0.9913	1.089	1.017–1.164
	5.000	88.79 ± 0.45^b^				
	2.500	72.70 ± 0.54^c^				
	1.250	55.03 ± 2.27^d^				
	0.625	35.63 ± 0.64^e^				
	0.313	14.15 ± 1.05^f^				
1% Phenazino-1-carboxylic acid SC	50.000	87.98 ± 0.34^a^	y = 0.6694x + 3.4942	0.9929	9.538	8.845–10.290
	25.000	72.97 ± 0.40^b^				
	12.500	53.80 ± 0.22^c^				
	6.250	38.99 ± 1.23^d^				
	3.125	26.31 ± 0.23^e^				
	1.563	10.55 ± 2.07^f^				
80% Ethylicin EC	120.000	99.44 ± 0.07^a^	y = 1.0102x + 2.5205	0.9918	11.802	11.105–12.525
	60.000	93.96 ± 0.12^b^				
	30.000	79.26 ± 0.27^c^				
	15.000	58.58 ± 0.70^d^				
	7.500	34.00 ± 1.44^e^				
	3.750	14.26 ± 0.66^f^				
5% Avermectin EC	160.000	69.22 ± 0.26^a^	y = 0.5817x + 2.545	0.9949	68.449	62.100–76.062
	80.000	51.95 ± 0.37^b^				
	40.000	37.89 ± 0.73^c^				
	20.000	25.04 ± 0.54^d^				
	10.000	14.89 ± 0.20^e^				
	5.000	5.56 ± 0.18^f^				
3% Zhongshengmycin AS	500.000	69.58 ± 0.83^a^	y = 0.2899x + 3.6878	0.9952	92.720	79.722–108.872
	250.000	61.73 ± 0.69^b^				
	125.000	51.60 ± 0.50^c^				
	62.500	44.97 ± 1.66^d^				
	31.250	38.04 ± 1.09^e^				
	15.625	30.89 ± 0.96^f^				
4% Berberine AS	500.000	68.69 ± 1.58^a^	y = 0.2884x + 3.6606	0.9929	104.104	88.786–122.769
	250.000	58.68 ± 0.69^b^				
	125.000	50.62 ± 0.82^c^				
	62.500	45.54 ± 0.97^d^				
	31.250	36.78 ± 0.69^e^				
	15.625	29.02 ± 0.43^f^				
8% Ningnanmycin AS	1000.000	81.81 ± 0.07^a^	y = 0.407x + 3.0393	0.9931	124.261	109.866–139.698
	500.000	70.68 ± 0.85^b^				
	250.000	60.22 ± 0.53^c^				
	125.000	47.97 ± 0.12^d^				
	62.500	39.37 ± 1.20^e^				
	31.250	30.27 ± 0.21^f^				

Different lowercase letters indicate significant differences in the inhibition rates of the same fungicide at different concentrations (*p* < 0.05). x represents the logarithm value of mass concentration, and y represents the probability of mycelial growth inhibition rate.

Among the seven biological pesticides, the inhibitory effect of 0.4% osthole SL on *F. ipomoeae* is the best, with an EC_50_ value of 1.089 μg/mL. 1% phenazino-1-carboxylic acid SC and 80% ethylicin EC show good inhibitory effect on mycelia growth, with EC_50_ values of 9.538 μg/mL and 11.802 μg/mL, respectively. Followed by 5% avermectin EC and 3% zhongshengmycin AS, with EC_50_ values of 68.449 μg/mL and 92.720 μg/mL, respectively. The inhibitory effect of 4% berberine AS and 8% ningnanmycin AS is poor, with EC_50_ values of 104.104 μg/mL and 124.261 μg/mL, respectively.

### 3.7 Potted plant prevention effect tests

**Figure 5:**
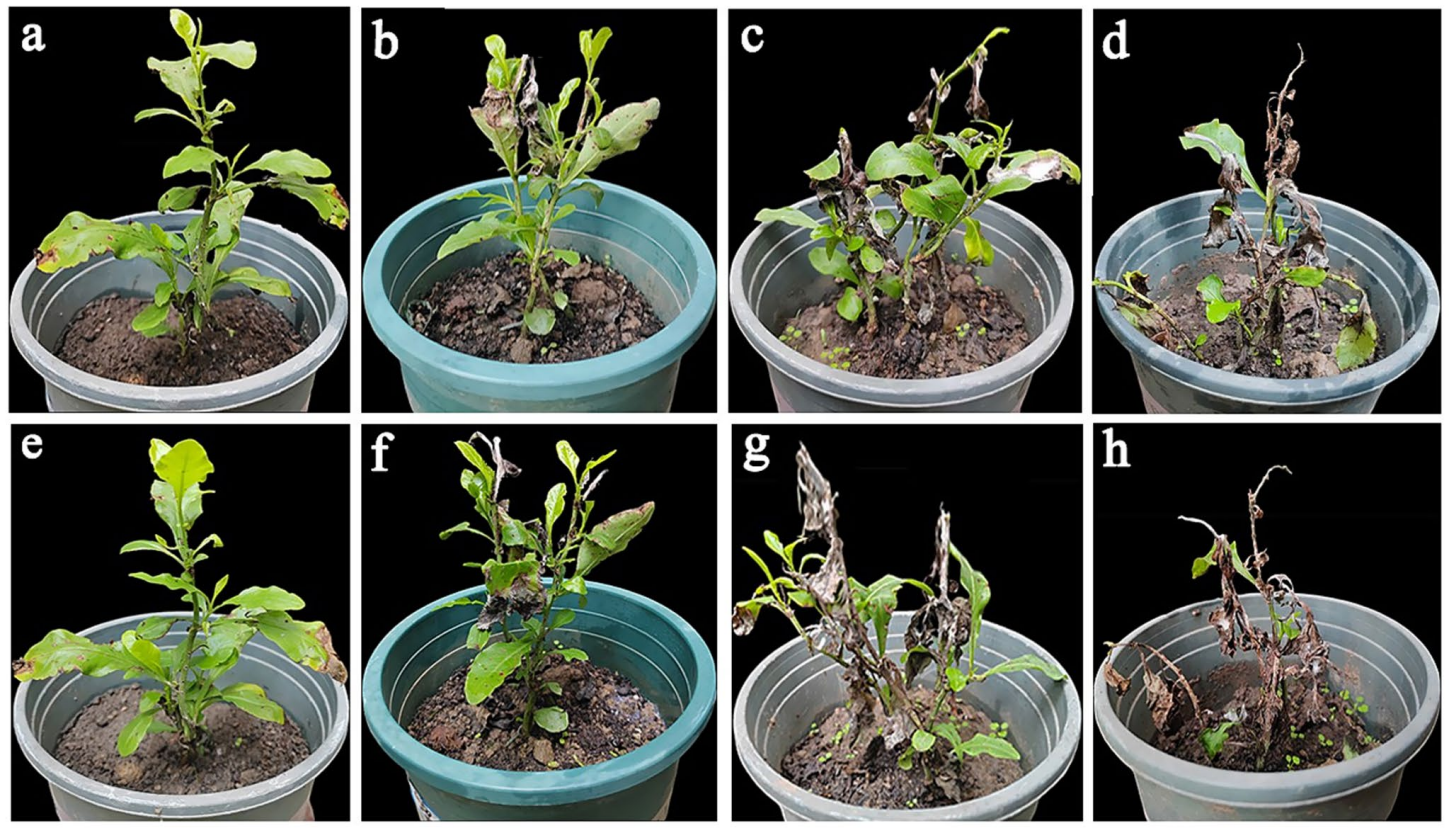
The potted control effect of osthole on *Plumbago auriculata* blight. **(a–c)** The control effect of potted plants before the fourth application of osthole (EC_90_, EC_70_, and EC_50_, respectively). **(d)** The control group before the fourth application of the osthole. **(e–g)** The control effect of potted plants 7 days after the fourth application of osthole (EC_90_, EC_70_, and EC_50_, respectively). **(h)** The control group was 7 days after the fourth application of the osthole.

The control effects of different concentrations of 0.4% osthole SL on the blight of *P. auriculata* potted plants caused by *F. ipomoeae* were evaluated (Table 5; Figure 5). The results showed that 0.4% osthole SL has a good control efficacy on *P. auriculata* blight caused by *F. ipomoeae*. However, three different concentrations of osthole treated had significantly different control efficacy on *P. auriculata* blight. Before the fourth application, 0.4% osthole SL had the highest control efficacy at a concentration of 5.244 μg/mL (EC_90_), reaching 91.10%, significantly higher than 2.071 μg/mL (EC_70_) and 1.089 μg/mL (EC_50_). After the 4th application for 7 days, the control efficacy remained good at a concentration of 5.244 μg/mL of 0.4% osthole SL, reaching 88.00%. In contrast, the control effects of 0.4% osthole SL concentrations of 2.071 μg/mL (EC_70_) and 1.089 μg/mL (EC_50_) were weaker, with 66.01 and 43.62%, respectively. 0.4% osthole SL can be used as a biological pesticide for the field control of *P. auriculata* blight caused by *F. ipomoeae*, and the recommended application concentration is 5.244 μg/mL.

**Table 5:** Control efficacy of osthole against blight of *Plumbago auriculata* by pot culture.

Treatment concentration/μg/mL	Before the fourth application		7 days after the fourth application	
	Disease severity index (DSI)	Control efficacy/%	Disease severity index (DSI)	Control efficacy/%
5.244 (EC_90_)	5.56 ± 0.42	91.10^a^	8.55 ± 0.31	88.00^a^
2.071 (EC_70_)	17.94 ± 0.34	71.30^b^	24.22 ± 0.66	66.01^b^
1.089 (EC_50_)	29.38 ± 0.59	52.99^c^	40.17 ± 0.72	43.62^c^
Control (CK)	62.50 ± 0.84	—	71.25 ± 0.95	—

The normal letters in the table indicate significant difference at the 0.05 level.

## 4 Discussion and conclusion

This study used morphological observation, a pathogenicity test, and molecular identification to identify the pathogen of *P. auriculata* blight disease. It was determined that the pathogen causing *P. auriculata* blight was *F. ipomoeae*. This is the first report of *F. ipomoeae* causing wilt on *P. auriculata* in China.

*Fusarium ipomoeae* is a member of the *Fusarium incarnatum-equiseti* species complex (FIESC). The holotype of *F. ipomoeae* was isolated from *Ipomoea aquatica* by L. Cai in 2016, named after the host genus, *Ipomoea* (Wang et al., 2019). *Fusarium ipomoeae* has a wide range of hosts and causes many plant disease symptoms after infection, causing leaf spot diseases on *Bletilla striata* (Zhou et al., 2020), peanut (Xu et al., 2021), tobacco (Wang et al., 2022) and sweet cherries (Zhou et al., 2022), and can also cause *Fusarium* wilt on soybean (Choi et al., 2022), maize leaf blight (Xu et al., 2022) and head blight of wheat (Al-Hashimi et al., 2025). In addition, *F. ipomoeae* can also cause rot in different parts of the plant. Rhizome rot of tobacco (Liu et al., 2022), crown rot of wheat (Ma et al., 2024), root rot of *Medicago sativa* (Suo et al., 2024), leaf rot on *Hosta plantaginea* (Zhou L. et al., 2023), fruit rot disease on *Cucumis melo* (Zhang et al., 2024), strawberry fruit rot (Li et al., 2023), fruit rot of *Cucurbita maxima* (Kitabayashi et al., 2023), pod rot of *Vigna mungo* (Verma et al., 2023).

At present, there are many types of fungicides for the control of plant diseases caused by *Fusarium* spp., but there are few studies on the screening of fungicides for *F. ipomoeae*. Suo et al. (2024) found that *F. ipomoeae* was most suitable for growth under the conditions of temperature 30°C, pH 6–10, and low nitrogen, and could withstand 150 g/L NaCl stress, showing strong environmental adaptability. Chemical fungicide fludioxonil had an obvious inhibitory effect on the colony growth of *F. ipomoeae*. Median effect concentration is 0.142 μg/mL. Compared with traditional chemical pesticides, biopesticides are highly selective, safe for humans and animals, safe for the natural ecological environment, and pollution-free. However, there are no reports on the screening of *F. ipomoeae* biopesticides. In this study, we evaluated the inhibitory effects of seven biopesticides on the growth of *F. ipomoeae*, a pathogen of *P. auriculata* blight. Through the study, the inhibitory effect of 0.4% osthole SL on *F. ipomoeae* was screened to be the most significant, with an EC_50_ value of 1.119 μg/mL. Osthole can reduce the activity of chitinase and *β*-1, 3-glucanase in *Fusarium*, to destroy the integrity and homeostasis of the *Fusarium* cell wall, thus inhibiting the normal growth of mycelia (Hu et al., 2023). Osthole has been proven to have a broad spectrum of antibacterial activity (Zhang et al., 2016). It is reported that osthole has a good inhibitory effect on growth on *Alternaria alternata* (Gu et al., 2024), *Erysiphe paeoniae* (Wu et al., 2023), *Cladobotryum asterophorum* (Yuan et al., 2023), *Nothophoma quercina* (Sun et al., 2024), *Puccinia helianthi* (Zhang et al., 2023), *Athelia rolfsii* (Wang et al., 2023). Some studies have reported that osthole has a good preventive and control effect on other plant diseases. Osthole has a good prevention and control effect on apple spotted leaf fall disease (Zhai et al., 2024), crown rot of wheat (Zhou F. et al., 2023), and gray mold of *Paris polyphylla* (Tang et al., 2021).

Although biopesticides are more environmentally friendly than chemical pesticides, they are not completely immune to the problem of drug resistance. Fungal drug resistance is an ongoing issue in both agriculture and medicine. Mechanisms of fungal drug resistance involve target alterations and non-target alterations. In Fusarium, the target alterations include generation of point mutations and overexpression of target genes, and the non-target alterations mainly involve drug efflux and biofilm formation (Zhao et al., 2021). Hengwei et al. (2018) observed that the Y137H mutation results in the resistance of *F. graminearum* to tebuconazole. The absence of CYP51C can increase the sensitivity of *F. graminearum* to tebuconazole and other drugs (Liu et al., 2011). In Fusarium, efflux pump research mainly focuses on ABC transporters, which use the energy generated by ATP hydrolysis to transport substances into or out of cell membranes (Locher, 2016). The ABC family in Fusarium regulates the resistance to azole drugs. Specifically, FcABC1 in *Fusarium culmorum* (Hellin et al., 2018), FgABC3, FgABC4 and FgABC-C9 in *Fusarium graminearum* are all related to the resistance of azole drugs (Abou Ammar et al., 2013; Qi et al., 2018).

Although this study screened out highly efficient and low-toxicity biological fungicides, repeated use of 0.4% osthole SL may select for resistant *F. ipomoeae* strains, compromising long-term disease management. Díaz-Gutiérrez et al. (2021) verified the fungicidal or fungistatic action of *Trichoderma asperellum* by sub-culturing the mycelia of the phytopathogenic fungi. In subsequent studies, it is necessary to conduct sub-culturing of pathogenic fungi. Monitor shifts in EC_50_ values across generations and analyze expression of resistance-associated genes. Future work remains essential to clarify the mechanisms involved in *F. ipomoeae* drug resistance and to develop more effective drugs.

## 5 Conclusion

This study identified the pathogenic fungi, *F. ipomoeae*, responsible for causing *P. auriculata* blight in Wanzhou District, Chongqing. This is the first report that *F. ipomoeae* causes blight on *P. auriculata* in China. *Fusarium ipomoeae* isolated from *P. auriculata* blight can infect other horticultural plants. And screen out biological fungicides that have inhibitory effects on *F. ipomoeae*. The efficacy of 0.4% osthole SL was tested under controlled greenhouse conditions. The results of this study provide a reference for the identification of *P. auriculata* blight and precise green control.

## Data Availability

The datasets presented in this study can be found in online repositories. The names of the repository/repositories and accession number(s) can be found in the article/supplementary material.

## Footnotes

^1^ http://www.ncbi.nlm.nih.gov

## References

[ref1] Abou Ammar G. Tryono R. Döll K. Karlovsky P. Deising H. B. Wirsel S. G. (2013). Identification of ABC transporter genes of *Fusarium graminearum* with roles in azole tolerance and/or virulence. PLoS One 8:e79042. doi: 10.1371/journal.pone.0079042, PMID: 24244413 PMC3823976

[ref2] Al-Hashimi A. Daniel A. I. Aina O. Du Plessis M. Keyster M. Klein A. (2025). Survey and identification of Fusarium head blight pathogens of wheat in the Western cape region of South Africa. Pathogens. 14:80. doi: 10.3390/pathogens14010080, PMID: 39861041 PMC11768704

[ref3] Ao X. Shi T. Yang W. Ouyang H. Fan R. Siddiqui J. A.. (2024). Biological characterization and *in vitro* fungicide screening of a new causal agent of walnut leaf spot in Guizhou province, China. Front. Microbiol. 15:487. doi: 10.3389/fmicb.2024.1439487, PMID: 39450284 PMC11500075

[ref4] Balamurugan A. Ashajyothi M. Shanu K. Charishma K. Varun H. Gunjeet K.. (2024). Chrysanthemum wilt caused by *Fusarium incarnatum*: etiology unveiled through polyphasic taxonomic methods. Physiol. Mol. Plant Pathol. 129:102214. doi: 10.1016/j.pmpp.2023.102214

[ref5] Choi H. W. Ryu H. Lee Y. Jang Y. W. Yi H. Hong S. K.. (2022). First report of *Fusarium ipomoeae* causing fusarium wilt on *Glycine max* in South Korea. Plant Dis. 107:575. doi: 10.1094/pdis-07-21-1499-pdn

[ref6] Díaz-Gutiérrez C. Arroyave C. Llugany M. Poschenrieder C. Martos S. Peláez C. (2021). *Trichoderma asperellum* as a preventive and curative agent to control Fusarium wilt in *Stevia rebaudiana*. Biol. Control 155:104537. doi: 10.1016/j.biocontrol.2021.104537

[ref7] Gu Q. Huang C. Wu Z. Zhang Y. Huang Y. Shen J. (2024). Identification,Biological characteristics and botanical pesticide screening of the Pathogenof black spot on *Paeonia lactiflora*. J Chin Med Mater. 2024, 2428–2433. doi: 10.13863/j.issn1001-4454.2024.10.004

[ref8] Hassan O. Ryu H. Choi H. W. (2023). First report of Verticillium wilt caused by *Verticillium dahliae* on chilli in South Korea. Plant Dis. 108:219. doi: 10.1094/pdis-07-23-1437-pdn

[ref9] Hellin P. King R. Urban M. Hammond-Kosack K. E. Legrève A. (2018). The adaptation of *Fusarium culmorum* to DMI fungicides is mediated by major transcriptome modifications in response to azole fungicide, including the overexpression of a PDR transporter (FcABC1). Front. Microbiol. 9:1385. doi: 10.3389/fmicb.2018.01385, PMID: 29997598 PMC6028722

[ref10] Hengwei Q. Juan D. Mengyu C. Xiaomei S. Wenxing L. Jinguang H.. (2018). The Y137H mutation in the cytochrome P450 FgCYP51B protein confers reduced sensitivity to tebuconazole in *Fusarium graminearum*. Pest Manag. Sci. 74, 1472–1477. doi: 10.1002/ps.4837 29274114

[ref11] Hu K. Li R. Mo F. Ding Y. Zhou A. Guo X.. (2023). Natural product osthole can significantly disrupt cell wall integrity and dynamic balance of *Fusarium oxysporum*. Pestic. Biochem. Physiol. 196:105623. doi: 10.1016/j.pestbp.2023.105623, PMID: 37945232

[ref12] Kitabayashi S. Kawaguchi A. Yoshida M. Kami D. Sugiyama K. Kawakami A. (2023). First report of *Fusarium ipomoeae* and *F. citri* causing postharvest fruit rot of winter squash (*Cucurbita maxima*). J. Gen. Plant Pathol. 89, 61–66. doi: 10.1007/s10327-022-01103-3

[ref13] Kumar S. Milton B. Mufeeda K. T. Singh R. (2025). *Paramyrothecium travancorense*: a novel fungal pathogen causing leaf spots and blights on *Coffea travancorensis* in Kerala, India. Physiol. Mol. Plant Pathol. 137:102609. doi: 10.1016/j.pmpp.2025.102609

[ref14] Kwon J.-H. Okhee C. Byeongsam K. Yeyeong L. Jiyeong P. Dong-Wan K.. (2017). Identification of *Lasiodiplodia pseudotheobromae* causing mango dieback in Korea. Can. J. Plant Pathol. 39, 241–245. doi: 10.1080/07060661.2017.1329231

[ref15] Li Z. Yu X. Zhang W. Han R. Zhang J. Ma Y.. (2023). Identification, characterization, and pathogenicity of fungi associated with strawberry fruit rot in Shandong Province, China. Plant Dis. 107, 3773–3782. doi: 10.1094/pdis-04-23-0696-re, PMID: 37408124

[ref16] Lin S. Liu Z. Guo J. Zhu L. Xu W. Wu W.. (2023). Identification of *Fusarium sacchari* causing foliar blight on *Begonia semperflorens* in China. Plant Dis. 107:3279. doi: 10.1094/pdis-01-23-0124-pdn, PMID:

[ref17] Liu X. Li Y. Cai L. Zhang C. Yin J. Wang H. (2022). Isolation and identification of pathogenic fungi from the rhizomes of tobacco. Chin. Tob. Sci. 43, 45–52. doi: 10.13496/j.issn.1007-5119.2022.06.007

[ref18] Liu X. Yu F. Schnabel G. Wu J. Wang Z. Ma Z. (2011). Paralogous cyp51 genes in *Fusarium graminearum* mediate differential sensitivity to sterol demethylation inhibitors. Fungal Genet. Biol. 48, 113–123. doi: 10.1016/j.fgb.2010.10.004, PMID: 20955812

[ref19] Locher K. P. (2016). Mechanistic diversity in ATP-binding cassette (ABC) transporters. Nat. Struct. Mol. Biol. 23, 487–493. doi: 10.1038/nsmb.3216, PMID: 27273632

[ref20] Lyu Y. Dong X. Sun H. Li R. Li S. Hu L.. (2024). First report of *Alternaria* leaf spot caused by *Alternaria alternata* on *Ulmus parvifolia* in Jiangsu, China. Plant Dis. 108:807. doi: 10.1094/pdis-10-23-2038-pdn, PMID:

[ref21] Ma G. Wang H. Qi K. Ma L. Zhang B. Zhang Y.. (2024). Isolation, characterization, and pathogenicity of *Fusarium* species causing crown rot of wheat. Front. Microbiol. 15:1405115. doi: 10.3389/fmicb.2024.1405115, PMID: 38873144 PMC11169711

[ref22] Mohamed R. A. Al-Bedak O. A. Hassan S. H. A. (2021). First record in upper Egypt of vascular wilt on pomegranate caused by *Fusarium oxysporum*, its molecular identification and artificial pathogenicity. J. Plant Dis. Prot. 128, 311–316. doi: 10.1007/s41348-020-00385-z

[ref23] Neupane S. Alexander L. Baysal-Gurel F. (2023). Evaluation of Hydrangea cultivars for tolerance against root rot caused by *Fusarium oxysporum*. Plant Dis. 107, 3967–3974. doi: 10.1094/pdis-11-22-2712-re, PMID: 37392028

[ref24] Ning H. Zhang J. Shao F. Wu X. Fan Y. (2011). Investigation on landscape application of blue flower plants in Hangzhou. J. Northwest For. Univ. 26, 173–176.

[ref25] O’Donnell K. Nirenberg H. I. Aoki T. Cigelnik E. (2000). A multigene phylogeny of the *Gibberella fujikuroi* species complex: detection of additional phylogenetically distinct species. Mycoscience 41, 61–78. doi: 10.1007/BF02464387

[ref26] O'Donnell K. Kistler H. C. Cigelnik E. Ploetz R. C. (1998). Multiple evolutionary origins of the fungus causing Panama disease of banana: concordant evidence from nuclear and mitochondrial gene genealogies. Proc. Natl. Acad. Sci. USA 95, 2044–2049. doi: 10.1073/pnas.95.5.2044, PMID: 9482835 PMC19243

[ref27] Panda P. Rihne T. Reddy M. G. Rao G. P. (2019). First report of the association of a ‘*Candidatus* Phytoplasma asteris’-related strain with *Plumbago auriculata* leaf yellowing in India. New Dis. Rep. 40:15. doi: 10.5197/j.2044-0588.2019.040.015

[ref28] Pandey A. K. Hubballi M. Sharma H. K. Ramesh R. Roy S. Dinesh K.. (2024). Molecular delineation and genetic diversity of *Fusarium* species complex causing tea dieback in India and their sensitivity to fungicides. Crop Prot. 181:106707. doi: 10.1016/j.cropro.2024.106707

[ref29] Promwee A. Yenjit P. Issarakraisila M. Intana W. Chamswarng C. (2017). Efficacy of indigenous *Trichoderma harzianum* in controlling *Phytophthora* leaf fall (*Phytophthora palmivora*) in Thai rubber trees. J. Plant Dis. Prot. 124, 41–50. doi: 10.1007/s41348-016-0051-y

[ref30] Qi P. F. Zhang Y. Z. Liu C. H. Zhu J. Chen Q. Guo Z. R.. (2018). *Fusarium graminearum* ATP-binding cassette transporter gene FgABCC9 is required for its transportation of salicylic acid, fungicide resistance, mycelial growth and pathogenicity towards wheat. Int. J. Mol. Sci. 19:351. doi: 10.3390/ijms19082351, PMID: 30103374 PMC6121456

[ref31] Reeb V. Lutzoni F. Roux C. (2004). Contribution of *RPB2* to multilocus phylogenetic studies of the euascomycetes (Pezizomycotina, Fungi) with special emphasis on the lichen-forming Acarosporaceae and evolution of polyspory. Mol. Phylogenet. Evol. 32, 1036–1060. doi: 10.1016/j.ympev.2004.04.012, PMID: 15288074

[ref32] Rui L. Zhang Q.-Q. Kong W.-L. Ni H. Wu X.-Q. (2025). First report of *Botryosphaeria dothidea* causing leaf blight on *Yulania denudata* in China. Crop Prot. 193:107180. doi: 10.1016/j.cropro.2025.107180

[ref33] Shen P. Gao S. Hu J. Li Y. Lei T. Shi L. (2021). In vitro flowering of the distylous plant *Plumbago auriculata* lam. S. Afr. J. Bot. 137, 492–498. doi: 10.1016/j.sajb.2020.11.018

[ref34] Soleha S. Muslim A. Suwandi S. Kadir S. Pratama R. (2022). The identification and pathogenicity of *Fusarium oxysporum* causing acacia seedling wilt disease. J. For. Res. 33, 711–719. doi: 10.1007/s11676-021-01355-3

[ref35] Sun Y. Jiao J. Li G. Guo T. Ding J. Guo M.. (2024). Identification, biological characteristics and sensitivity to different fungicides of the pathogen causing stem canker of *Pinus bungeana*. J Northwest Forest Univ. 39, 18–25+66. doi: 10.3969/j.issn.1001-7461.2024.03.03

[ref36] Suo X. Niu Q. Guo C. Gai Y. Chen L. Yin S. (2024). Isolation of a novel alfalfa *Fusarium* root rot pathogen FIESC and characterization of strain biology. Acta Agrestia Sin. 32, 1327–1338. doi: 10.11733/j.issn.1007-0435.2024.05.003

[ref37] Tang T. Wang F. Duan Y. Guo X. Guo J. You J. (2021). Control effect of thirteen biological fungicides on the graymold of *Paris polyphylla*. Agrochemical. 60, 297–300. doi: 10.16820/j.cnki.1006-0413.2021.04.017

[ref38] Tang X. Yangjing G. Zhuoma G. Guo X. Cao P. Yi B.. (2022). Biological characterization and *in vitro* fungicide screenings of a new causal agent of wheat Fusarium head blight in Tibet, China. Front. Microbiol. 13:734. doi: 10.3389/fmicb.2022.941734, PMID: 35992662 PMC9389214

[ref39] Verma R. Kushwaha K. P. S. Chakrawarti N. Kumar S. Kaur M. Gupta P. K.. (2023). First report of *Fusarium incarnatum-equiseti* species complex as the causal agent of pod rot of black gram (*Vigna mungo*) in India. Plant Dis. 107:2855. doi: 10.1094/pdis-02-23-0363-pdn, PMID:

[ref40] Vyapari S. Scheiber S. M. Thralls E. L. (2007). Pre-transplant root ball condition influences growth of Plumbago during establishment. HortTechnology. 17, 486–490. doi: 10.21273/HORTTECH.17.4.486

[ref41] Wang M. M. Chen Q. Diao Y. Z. Duan W. J. Cai L. (2019). *Fusarium incarnatum-equiseti* complex from China. Persoonia 43, 70–89. doi: 10.3767/persoonia.2019.43.03, PMID: 32214498 PMC7085858

[ref42] Wang Q. Huang J. Li S. Zhang Y. Li Z. Liu Y.. (2024). Identification of the pathogen of sorghum anthracnose in Chongqing and fungicides screening. Plant Prot. 50, 323–330+367. doi: 10.16688/j.zwbh.2023458

[ref43] Wang C. X. Hulei Z. Shenhai W. Shengfeng M. (2021). Leaf spot of *Hosta ventricosa* caused by *Fusarium oxysporum* in China. PeerJ. 9:e12581. doi: 10.7717/PEERJ.12581 34966590 PMC8663626

[ref44] Wang H. Li Y. Li W. Cai L. Meng J. Xia G.. (2022). Morphological and molecular identification of *Fusarium ipomoeae* as the causative agent of leaf spot disease in tobacco from China. Microorganisms. 10:1890. doi: 10.3390/microorganisms10101890, PMID: 36296167 PMC9611381

[ref45] Wang F. Tang T. He Y. Guo X. Duan Y. You J. (2023). Identification, biological characterisation, and fungicides screening of pathogen causing southern blight of *Coptis chinensis* in Lichuan city. Act Phytopathol Sin. 53, 789–795. doi: 10.13926/j.cnki.apps.000846

[ref46] White T. J. Bruns T. Lee S. Taylor J. (1990). Amplification and direct sequencing of fungal ribosomal RNA genes for phylogenetics. eds. M. A. Innis, D. H. Gelfand, J. J. Sninsky and T. J. White, PCR Protocols. 315–322. doi: 10.1016/B978-0-12-372180-8.50042-1, PMID: 40544123

[ref47] Wu Z. Zhang Y. Chang H. Yang K. Xu Z. Wang L.. (2023). Pathogen identification and field efficacy test of powdery mildew on *Paeonia lactiflora* in Hexi area of Gansu Province. Agrochemiaals. 62, 758–762+776. doi: 10.16820/j.nyzz.2023.1045

[ref48] Xu X. Dai T. Wu C. (2024). First report of *Fusarium vanettenii* causing fusarium root rot in *Fatsia japonica* in China. Forests 15:805. doi: 10.3390/f15050805

[ref49] Xu X. Zhang L. Yang X. Shen G. Wang S. Teng H.. (2022). *Fusarium* species associated with maize leaf blight in Heilongjiang Province, China. J Fungi. 8:170. doi: 10.3390/jof8111170, PMID: 36354937 PMC9698036

[ref50] Xu M. Zhang X. Yu J. Guo Z. Li Y. Wu J.. (2021). First report of *Fusarium ipomoeae* causing peanut leaf spot in China. Plant Dis. 105:3754 doi: 10.1094/pdis-01-21-0226-pdn

[ref51] Yang L. Gao W. Zhang C. Huo J. Wang Y. (2023). Identification of strawberry wilt caused by *Plectosphaerella cucumerina* in China. Plant Dis. 107:3290. doi: 10.1094/pdis-03-23-0544-pdn

[ref52] Yao L. Ji C. Wang Z. Liu Y. Li W. Wang M.. (2025). Enhancing *Astragalus mongholicus* performance through endophytic fungi improvement for *Fusarium* wilt resistance. Physiol. Mol. Plant Pathol. 136:102532. doi: 10.1016/j.pmpp.2024.102532

[ref53] Yuan X. Peng K. Zhao Z. Wang Y. Tian F. Li Y. (2023). Identification of the pathogen causing cobweb disease on black termite mushroom *Hymenopellis raphanipes* in Guizhou Province, with analysis of its biologicalcharacteristics and screening of fungicides for its control. J. Plant Prot. 50, 780–790. doi: 10.13802/j.cnki.zwbhxb.2023.2021145

[ref54] Zhai Y. Zheng G. Li J. Xu B. Zhang Z. Hui N.. (2024). The control effects of 12 kinds of biogenic agents on apple spot leaf drop disease(*Alternaria alternata*). Acta Agric. Boreali-Occid. Sin. 33, 1559–1566. doi: 10.7606/j.issn.1004-1389.2024.08.017

[ref55] Zhang A. Lu Y. Huang J. Jing L. (2023). Control effects of 9 botanical fungicides on *Puccinia helianthi*. Chin. J. Oil Crop Sci. 45, 623–628. doi: 10.19802/j.issn.1007-9084.2022090

[ref56] Zhang S. L. Wang J. J. Shi C. Shi Z. R. Luan F. (2024). First report of fruit rot disease on melon (*Cucumis melo*) caused by *Fusarium ipomoeae* in China. Plant Dis. 108:2923. doi: 10.1094/pdis-02-24-0386-pdn, PMID:

[ref57] Zhang M. Z. Zhang R. R. Wang J. Q. Yu X. Zhang Y. L. Wang Q. Q.. (2016). Microwave-assisted synthesis and antifungal activity of novel fused Osthole derivatives. Eur. J. Med. Chem. 124, 10–16. doi: 10.1016/j.ejmech.2016.08.012, PMID: 27565553

[ref58] Zhao B. He D. Wang L. (2021). Advances in *Fusarium* drug resistance research. J. Glob. Antimicrob. Resist. 24, 215–219. doi: 10.1016/j.jgar.2020.12.016 33460843

[ref59] Zhou L. He W. Long Y. Li Y. Ma W. Yin F.. (2023). First report of *Fusarium ipomoeae* causing leaf rot on *Hosta plantaginea* in China. Plant Dis. 108:227. doi: 10.1094/pdis-09-23-1749-pdn

[ref60] Zhou F. Luo A. Han A. Li G. Xu L. Zhang F.. (2023). Study on the antibacterial activity of eight botanical fungicides against *Fusarium pseudograminearum* and control effects on *Fursarium* crown rot of wheat. J. Triticeae Crops. 43, 1629–1635. doi: 10.7606/jissn.1009-10412023.12.15

[ref61] Zhou L. Y. Yang S. F. Wang S. M. Lv J. W. Wan W. Q. Li Y. H.. (2020). Identification of *Fusarium ipomoeae* as the causative agent of leaf spot disease in *Bletilla striata* in China. Plant Dis. 105:1214. doi: 10.1094/pdis-09-20-1974-pdn

[ref62] Zhou Y. Zhang W. Li X. Ji S. Chethana K. W. T. Hyde K. D.. (2022). *Fusarium* species associated with cherry leaf spot in China. Plants. 11:760. doi: 10.3390/plants11202760, PMID: 36297784 PMC9609575

